# Pharmacological interventions in acute respiratory distress syndrome

**DOI:** 10.1186/2110-5820-3-20

**Published:** 2013-07-03

**Authors:** Antoine Roch, Sami Hraiech, Stéphanie Dizier, Laurent Papazian

**Affiliations:** 1 URMITE, UM63, CNRS 7278, Aix Marseille Université, IRD 198, Inserm 1095, Marseille, 13005, France; 2 APHM, CHU Nord, Réanimation, Marseille, 13015, France; 3 Intensive Care Unit, CHU Nord, Chemin des Bourrely, Marseille, 13015, France

**Keywords:** Acute respiratory distress syndrome, Nitric oxide, Neuromuscular blockers, Fluids, Albumin, Corticosteroids, Hypoxemia, Fibrosis, Rescue therapy, Outcome

## Abstract

Pharmacological interventions are commonly considered in acute respiratory distress syndrome (ARDS) patients. Inhaled nitric oxide (iNO) and neuromuscular blockers (NMBs) are used in patients with severe hypoxemia. No outcome benefit has been observed with the systematic use of iNO. However, a sometimes important improvement in oxygenation can occur shortly after starting administration. Therefore, its ease of use and its good tolerance justify iNO optionally combined with almitirne as a rescue therapy on a trial basis. Recent data from the literature support the use of a 48-h infusion of NMBs in patients with a PaO_2_ to FiO_2_ ratio <120 mmHg. No strong evidence exists on the increase of ICU-acquired paresis after a short course of NMBs. Fluid management with the goal to obtain zero fluid balance in ARDS patients without shock or renal failure significantly increases the number of days without mechanical ventilation. On the other hand, patients with hemodynamic failure must receive early and adapted fluid resuscitation. Liberal and conservative fluid strategies therefore are complementary and should ideally follow each other in time in the same patient whose hemodynamic state progressively stabilizes. At present, albumin treatment does not appear to be justified for limitation of pulmonary edema and respiratory morbidity. Aerosolized β2-agonists do not improve outcome in patients with ARDS and one study strongly suggests that intravenous salbutamol may worsen outcome in those patients. The early use of high doses of corticosteroids for the prevention of ARDS in septic shock patients or in patients with confirmed ARDS significantly reduced the duration of mechanical ventilation but had no effect or even increased mortality. In patients with persistent ARDS after 7 to 28 days, a randomized trial showed no reduction in mortality with moderate doses of corticosteroids but an increased PaO_2_ to FiO_2_ ratio and thoracopulmonary compliance were found, as well as shorter durations of mechanical ventilation and of ICU stay. Conflicting data exist on the interest of low doses of corticosteroids (200 mg/day of hydrocortisone) in ARDS patients. In the context of a persistent ARDS with histological proof of fibroproliferation, a corticosteroid treatment with a progressive decrease of doses can be proposed.

## Review

### Introduction

Dysregulated inflammation, accumulation and activity of leukocytes and platelets, uncontrolled activation of coagulation and altered permeability of alveolar endothelial and epithelial barriers leading to pulmonary edema are key points in the pathophysiology of acute lung injury and acute respiratory distress syndrome (ARDS) [1]. ARDS results in impaired gas exchange and hypoxemic respiratory failure, which can be refractory and life-threatening [2]. Besides ventilatory support, pharmacological interventions are commonly considered throughout the course of ARDS. Some of these interventions affect supportive care, such as fluid management or neuromuscular blockers (NMBs), whereas others are focused on recovery from hypoxemia or have been studied in patients with persistent ARDS. The present review will summarize clinical results and practical aspects of pharmacological interventions, which are more largely used by clinicians. The current evidence about inhaled nitric oxide, almitrine, NMBs, fluid management, β2-agonists, and corticosteroids will be addressed successively.

### Inhaled nitric oxide and almitrine

Inhaled nitric oxide (iNO) selectively vasodilates the pulmonary vasculature in ventilated alveoli, which improves ventilation-perfusion matching and hypoxemia and lowers pulmonary arterial pressure. Its administration via the inhaled route and its very short half-life minimize systemic effects (i.e., hypotension) [3]. In randomized, clinical trials (RCTs) [4-8], iNO was associated with a transient improvement in oxygenation in adults with ARDS. However, no survival benefit or reduction in ventilator-free days has been observed. In a systematic review and meta-analysis of patients with acute lung injury (ALI) or ARDS from 12 trials, iNO was associated with modest improvements in oxygenation (13% of increase in PaO_2_ to FiO_2_ ratio until day 3-4 of administration), no effect on mean pulmonary artery pressure, and no effect on survival or duration of mechanical ventilation [9]. However, available RCTs do not resolve the question of whether iNO leads to any clinically significant benefits in certain subgroups of patients, such as those with severe hypoxemia not responding to conventional treatment [10]. In this sense, iNO is frequently used as a rescue therapy. For example, iNO was used during ICU stay in respectively 30% and 20% of ARDS patients included in two large RCTs [11,12]. Moreover, iNO was used for refractory hypoxemia in 32% of the patients subsequently treated with ECMO during the 2009 H1N1 pandemic in Australia/New Zealand [13]. When defining a positive response as an increase of at least 20% of PaO_2_ to FiO_2_ ratio, about half of patients are responders to iNO [6,14]. However, because improvement in oxygenation can be dramatic in the setting of life-threatening hypoxemia and because no radiological or physiological predictive factor of response has been identified [15,16], its use can be supported in this setting on a trial basis, what may buy time for the institution of other means of support. Clinically significant improvement in oxygenation following initiation of iNO should be demonstrated within the first hour of therapy to justify continued use. A maximal oxygenation benefit typically occurs with doses as low as 0.1 to 2 ppm [17] and always with doses of less than 20 ppm [18]. Worsening of oxygenation has been frequently documented with doses above 20 ppm [19] or even above 10 ppm [18]. Moreover, the dose-response curve has been shown to shift progressively to the left in patients receiving iNO continuously for several days [20], implying that the optimal dose of iNO must be determined by titration against the therapeutic target in each patient at least each day. INO has additive effects on oxygenation with prone positioning [21] and with high frequency oscillatory ventilation [22]. The effect of iNO is not influenced by the administration of vasopressive agents, such as norepinephrine [23,24], but iNO has additive effects on oxygenation with almitrine, a selective pulmonary vasoconstrictor [25,26]. A progressive iNO weaning on several hours is suggested due to the risk of oxygenation worsening, pulmonary artery pressure increase, and hemodynamic collapse in case of sudden discontinuation [14]. INO may contribute to the formation of cytotoxic reactive nitrogen species and reactive oxygen species, especially when administered with high concentrations of oxygen [27]. This is minimized by limiting FiO_2_, by introducing iNO into the inspiratory limb of the ventilator tubing as near to the patient as possible [28] and by synchronizing injection of iNO with inspiration [29]. Up to 40 ppm of iNO should not cause methemoglobinemia in adults in the absence of methemoglobin reductase deficiency [30].

Experimentally, almitrine increases hypoxic pulmonary vasoconstriction, except where strong vasoconstriction is already present [31]. In patients with ARDS, almitrine causes a redistribution of blood flow to well-ventilated areas and improves oxygenation [32] while increasing pulmonary artery pressure [33-35]. It is suggested that low-dose almitrine enhances hypoxic pulmonary vasoconstriction, whereas at high doses (>20 μg/kg/min), it would induce diffuse and inefficient vasoconstriction [36,37]. In this sense, dosages of 2 to 4 μg/kg/min have been shown to be optimal in most patients [34,38,39]. Almitrine has additive effects on oxygenation with iNO in approximately two thirds of iNO responders [25,38]. However, some patients may be responders to almitrine while they did not respond to iNO [25,38]. The effectiveness of almitrine on oxygenation often is reduced in septic patients receiving norepinephrine [23,24]. Therefore, the use of higher doses of almitrine (16 μg/kg/min) may produce further improvement in oxygenation in patients receiving norepinephrine if they are responders to iNO [26]. The efficiency of prolonged administration of almitrine has not been evaluated. Because of an increased pulmonary artery pressure, high doses of almitrine (16 μg/kg/min) may result in reduced right ventricular ejection fraction [40]. Therefore, almitrine is not recommended in patients with severe pulmonary hypertension or acute cor pulmonale. The prolonged administration of almitrine has been involved in peripheral neuropathies in COPD patients [41,42]. This complication was usually associated with circulating levels >400 ng/ml. While intravenous administration of 16 μg/kg/min of almitrine in patients with ARDS was associated with levels >400 ng/ml in 16 of 17 patients, a dosage of 4 μg/kg/min resulted in high levels in only one patient [32], suggesting that this dosage would be safe on the neurological function. The almitrine also may have liver toxicity [43]. A reversible and dose-dependent increase in lactate level associated with impaired liver function was observed in 8 of 25 patients in the first 24 hours of administration of 2 of 8 μg/kg/min of almitrine [43]. As for iNO, a frequent and quick effect on oxygenation supports the optional use of almitrine in patients with very severe hypoxemia on a trial basis, until the institution of other means of support. The absence of response to the administration of 0.5 mg/kg in 30 minutes does not justify any continued administration.

### Neuromuscular blockers

Several studies have reported the frequent use of neuromuscular blockers (NMBs) in severe ARDS patients, reaching 40-50% in several recent RCTs [12,44,45]. One of the main reasons to justify the use of NMBs in ARDS is facilitation of mechanical ventilation and control of patient/ventilator asynchrony. Arroliga et al. [46] found that the factors associated with a more frequent NMB use were mainly related to the severity of the lung disease, such as the presence of ARDS criteria, high plateau pressures, and also by the use of prone positioning, high PEEP levels, or nonconventional modes of ventilation, such as high-frequency oscillatory ventilation [47]. Several recent studies have shown an improvement in oxygenation with the use of NMBs in ARDS patients [11,48-50]. In the studies by Gainner et al. [49] and Forel et al. [50], patients randomized to the NMB group had a higher PaO_2_ to FiO_2_ ratio at 48, 96, and 120 h after randomization. In contrast, there was no change in the PaO_2_ to FiO_2_ ratio 1 h after randomization in the NMB group. The decrease in plateau pressure and PEEP requirements during the 120-h study period were more marked in the NMB group. Recently, a larger RCT confirmed these results [11], showing a PaO_2_ to FiO_2_ ratio on day 7 higher in patients receiving a 48-h continuous cisatracurium infusion than in the control group. In this multicenter, double-blind trial [11], 339 patients presenting with severe ARDS within the previous 48 h (i.e., PaO_2_ to FiO_2_ ratio <150 mmHg with PEEP of at least 5 cm H_2_O) were randomly assigned to receive either cisatracurium besylate (bolus of 15 mg i.v., then 37.5 mg/h infusion for 48 h) or placebo for 48 h. The group of patients treated with cisatracurium had a higher adjusted 90-day survival rate compared with those who received placebo (hazards ratio (HR) 0.68; 95% confidence interval (CI) 0.48–0.98; *p* = 0.04). It is noteworthy that the beneficial effect of cisatracurium on mortality was limited to the patients presenting a PaO_2_ to FiO_2_ ratio of less than 120 mmHg. These results were recently confirmed in a meta-analysis [51]. The observed improvements in mortality and gas exchange raise the question of the mechanisms involved. The positive effects of NMBs could be related to a decrease in ventilator induced lung injury, as suggested by the decreased incidence of barotrauma and pneumothoraces in the cisatracurium group [11]. NMBs also could reinforce the beneficial effect of lung-protective mechanical ventilation in patients with ARDS through a reduction in biotrauma. Forel et al. [50] showed that after 48 h of NMB infusion in ARDS patients, pulmonary concentrations of IL-1b, IL-6, and IL-8, as well as serum concentrations of IL-1b and IL-6 were lower than in the control group. This hypothesis is reinforced by the observed decrease in the number of organ failures in the cisatracurium group of the ACURASYS study [11], possibly as a result of less biotrauma. NMBs also could help to avoid patient–ventilator dyssynchrony and limit end-expiratory collapse by inhibiting active expiration, limiting derecruitment, and maintaining PEEP [52]. Moreover, in some patients, inspiratory efforts could lead to global or regional increases in transpulmonary pressure that can be deleterious [53]. These recent data from the literature provide a strong argument for beneficial effects of NMBs during the early phase of severe ARDS and support the use of a 48-h infusion of cisatracurium in patients with more hypoxemic ARDS (particularly with a PaO_2_ to FiO_2_ ratio <120 mmHg). However, risks of using NMBs have been reported and have resulted in controversy regarding the use of these agents in ARDS patients. The incidence of ICU-acquired weakness is 34–60% in patients with ARDS [54]. Administration of NMBs does not appear to be an independent risk factor for ICU-acquired weakness [55] if they are not given with corticosteroids or in patients with hyperglycemia [56]. In the ACURASYS study [11], the incidence of ICU-acquired paresis was not higher in patients receiving NMBs than in the control group. Hypersensitivity reactions occurring after administration of NMBs are a major cause for concern. A recent survey [57] of hypersensitivity reactions observed during anesthesia showed that cisatracurium was involved in 5.9% of anaphylaxis episodes. Finally, paralyzing patients highlights the problem of inadequate sedation [58].

### Fluid management

In the early phase of ARDS, an associated septic state is usually responsible for hypovolemia. At this stage, hemodynamic optimization by early and adapted fluid loading has proven its prognostic value [59] and a fluid restriction strategy can result in hemodynamic aggravation and dysfunctions of associated organs, determining the mortality of patients presenting ARDS [60]. Nevertheless, ARDS is particularly characterized by pulmonary edema due to an increase in pulmonary capillary permeability. Edema has an impact on respiratory function at several levels: decrease in compliance, hypoxemia, alteration of surfactant, pulmonary hypertension [61]. Thus, any attempt to reduce edema potentially can have beneficial effects on respiratory function and eventually outcome. Several studies have suggested a prognostic role of the quantity of lung water in ARDS patients [62-64]. Moreover, large cohort studies have shown that positive fluid balance was associated with higher mortality in patients with acute lung injury or ARDS [65] or in patients with septic shock [66]. However, to date, no study evaluated strategies specifically designed to reduce lung water content and to evaluate their prognostic impact. Fluid restriction that is more or less associated with a diuretic treatment makes possible to reduce both pulmonary capillary pressure and central venous pressure (CVP) due to lung edema resorption through an improvement in venous return. A randomized, multicenter study evaluated a strategy of fluid restriction that was more or less associated with diuretic treatment prescribed in the absence of hypotension and renal failure in patients with acute lung injury or ARDS [67]. Patients were included in the study approximately 48 hours after admission to the ICU. The decision to submit patients to fluid loading or diuretic treatment depended on the presence of oliguria and the level of CVP. Schematically, the purpose was to obtain a CVP of 8 mmHg or less in the “conservative-strategy” group or 14 mmHg in the “liberal-strategy” group. The protocol was applied for 7 days after inclusion of the patient but was not applied in cases of hypotension. The conservative strategy resulted in zero fluid in 7 days, whereas the fluid results in the liberal-strategy were +6 liters over 7 days. The conservative strategy discreetly improved oxygenation in patients and increased the number of days without ventilation (14.6 ± 0.5 vs. 12 ± 0.5, *p* < 0.001) but did not influence mortality at 60 days, which was the principal purpose of the study.

This study confirmed the impression that limiting the fluid intake of patients with isolated respiratory failure can limit respiratory morbidity without aggravating other organ dysfunctions. However, the exclusion of hemodynamically unstable patients or patients with renal failure makes impossible to generalize these results or to create a “gold standard” for management of the fluid status of every ARDS patient. In addition, the absence of an effect on mortality reminds us that the management of fluid intake in ICU patients does not boil down to being liberal or conservative. It is only once the initial phase of hemodynamic instability has passed that a reasonable policy of fluid intake aimed at zero fluid can contribute to reducing the duration of ventilation and ICU stay [68]. The importance of a “biphase” fluid strategy was recently illustrated by a retrospective study that included 212 patients presenting acute lung injury complicating septic shock [69].

In this study, the nonperformance of early adapted fluid administration and the absence of a negative fluid balance during a minimum of the first 2 consecutive days within the 7 days following the occurrence of septic shock were independent mortality factors in multivariate analysis. Along these lines, the Surviving Sepsis Campaign [70] recommends a conservative fluid strategy for patients with established ARDS who do not have evidence of tissue hypoperfusion. From a hemodynamic point of view, in case of a decrease in plasmatic oncotic pressure, which is clinically illustrated by hypoprotidemia/hypoalbuminemia, pulmonary edema forms at a lower hydrostatic pressure because the oncotic pressure gradient between plasma and the interstitium decreases [71]. Therefore, hypoprotidemia frequently observed in ICU patients facilitates the development of hydrostatic pulmonary edema. However, the importance of oncotic pressure in the limitation of flux is only conceivable if the barrier is intact. In case of endothelial lesions, an interstitial edema will be all the richer in proteins than the plasma, theoretically limiting the interest of increasing the plasmatic oncotic pressure [72]. The interest of a strategy of diuretic treatment associated with albumin filling in ARDS patients with a protidemia inferior to 50 g/l has been investigated by Martin et al. [73,74]. In the most recent study [74], 40 patients were randomized receiving either furosemide alone or furosemide and albumin (75 g/d) for 3 days. When protidemia was superior to 80 g/l in the treated group, albumin was replaced with a placebo. Albuminemia increased by 13 g/l in the albumin + furosemide group, reaching 30 g/l at the end of treatment and increased by 3 g/l in the furosemide alone group, reaching 20 g/l at the end of treatment. The effects can be summarized by a discrete improvement in oxygenation when albumin was associated with diuretic treatment compared with diuretic treatment alone.

A large, randomized study [75] demonstrated that volume therapy using albumin was equivalent to volume therapy using saline in ICU patients. At this time, the very limited clinical data do not make it possible to recommend the administration of albumin with the goal to improve pulmonary function and respiratory morbidity in ARDS patients. Two recent RCTs evaluated the effects of albumin in septic patients [76,77]. In the first, conducted in France [76], 798 patients with septic shock were randomized to receive either 300 ml of 20% albumin per day for 3 days or the same amount of saline. Neither mortality at day 28 nor the respiratory outcome was different between groups. The second was conducted in Italy [77] and included 1,800 patients with severe sepsis or septic shock. Patients were randomized to receive crystalloids and albumin to maintain albumin level >30 g/l until day 28 or crystalloids alone. Although day-90 mortality was not different between groups, the use of albumin in the subgroup of patients with septic shock was associated with a reduction of mortality.

No large study investigated the interest of using hydroxyethylstrach (HES) for fluid loading in ARDS patients. However, considering the increased risk of renal dysfunction associated with their use [78], especially in septic patients [79], they cannot be recommended in ARDS patients. The use of hyperosmolar filling solutions, such as hypertonic saline, could have the advantage, due to the limited amount of fluid administered, of limiting the development of pulmonary edema in case of an increase in the alveolocapillary barrier. Experimental studies have provided conflicting results on this point [80-82]. In clinical practice, the potential interest of hypertonic resuscitation has been investigated in patients with traumatic hypovolemic shock. In a recent randomized study that included 853 patients, fluid loading with hypertonic saline or hypertonic saline/dextran neither reduced mortality nor prevented ARDS occurrence compared with normal saline resuscitation [83].

### β2-agonists

Recovery from ARDS requires that the pulmonary edema resolve [84]. The resolution of alveolar edema is driven by active transport of sodium and chloride ions from the luminal space across alveolar epithelial cells, creating an osmotic gradient for the reabsorption of water. Despite severe epithelial lesions, alveolar clearance is usually pharmacologically stimulable. Several experimental studies have shown that the exogenous administration of cAMP agonists, in particular β2-agonists, accelerates the resolution of edema, whatever its nature [85]. A preliminary study [86] showed that the intravenous administration of salbutamol at a dose of 15 μg/kg/h for 7 days in ARDS patients made possible to diminish the quantity of pulmonary water measured by transpulmonary thermodilution without affecting oxygenation, duration of mechanical ventilation, or outcome. However, the same authors showed in a large RCT involving 326 patients [87] that 15 μg/kg/h of intravenous salbutamol was poorly tolerated and was associated with an increase in 28-day mortality (34 vs. 23% in the control group, *p* = 0.033). Another RCT has evaluated the effect of aerosolized albuterol six times per day until day 10 or extubation in patients with acute lung injury [88]. The study was stopped for futility after the inclusion of 282 patients. The number of ventilator-free days, which was the primary outcome variable, was not different between groups. These data do not support the use of aerosolized β2-agonists to improve outcome in patients with ARDS, and strongly suggest that intravenous salbutamol may worsen outcome in those patients.

### Corticosteroids

Corticosteroids have a broad inhibitory action on host defenses, including inhibition of the transcription of proinflammatory cytokines, inhibition of neutrophil activation, a synergic action with anti-inflammatory cytokines, and a suppression of the synthesis of phospholipase A2, cyclooxygenase, and inducible NO synthase [89]. Because of these properties, corticosteroids in high doses for a short period were proposed in the early phase of ARDS, mostly in septic patients, to obtain a quick resolution and to prevent progression to lung fibrosis.

Four randomized trials investigated early use of high doses of corticosteroids for the prevention of ARDS in septic shock patients [90-92] or in patients with confirmed ARDS [93]. In these studies, corticosteroids significantly reduced the duration of mechanical ventilation. However, these studies showed no benefit in terms of prevention or improvement of ARDS and no effect or even an increase of mortality with corticosteroids. Only relevant ARDS etiologies (pneumocystis pneumonia, pulmonary eosinophilic pneumonia associated with certain autoimmune diseases, or a systemic disease) are recognized as eligible for steroids in the early stages. However, some authors argue that the failure of steroids is not related to their early use but to a too short administration [94]. Corticosteroids have an inhibitory effect on fibroblast proliferation and collagen deposition, including in the lung. Therefore, they were proposed in the late phase of fibroproliferation and post-aggressive fibrosis. Several case reports and a small, randomized study suggested an interest in terms of reduced mortality [95]. This study, although on a small number of patients and despite methodological bias has long been the benchmark to justify corticosteroids in persistent ARDS.

More recently, another RCT by Meduri evaluated early corticosteroid administration, and showed that methylprednisolone administration (1 mg/kg/d) less than 72 after the onset of ARDS reduced mortality [96]. However, these results should be interpreted with caution, because this study included a large number of patients with septic shock. Another randomized, controlled trial, conducted by the ARDS network, showed no reduction in mortality in the group receiving methylprednisolone [44]. This study involved 180 patients with ARDS persistent after 7 to 28 days who received methylprednisolone (2 mg/kg/d) or placebo for 21 days. The protocol used was borrowed from corticosteroid Meduri study [95] and also is the protocol commonly used in clinical practice. Despite the lack of benefit in terms of mortality at 60 days, an increased PaO_2_ to FiO_2_ ratio and thoracopulmonary compliance were found in the group receiving corticosteroids, as well as shorter durations of mechanical ventilation and ICU stay. The subgroup analysis showed a significantly higher mortality rate when corticosteroid therapy was started after the 13th day of ARDS, suggesting the presence of fixed fibrotic lesions at this stage, at the expense of a steroid-sensitive fibroproliferation. However, this study had some bias, including a very important initial selection (5% of eligible patients included) with an only 30% mortality rate in persistent ARDS, a brutal discontinuation of steroids after 48 hours of ventilator weaning and finally the absence of pathologic analysis leading probably to a treatment with corticosteroids in some patients without fibroproliferation. The subgroup analysis suggests this hypothesis. Indeed, patients with a low level of procollagen III in their alveolar lavage fluid, thus having a lesser degree of fibroproliferation, had a higher mortality if they had received steroids (35% vs. 9%, *p* < 0.05) [44]. On the other hand, it is conceivable that some of them were carrying a contraindication to steroids because of an infection.

Papazian et al. [97] recently showed that only 53% of persistent ARDS patients actually had fibrosis or fibroproliferation highlighted on surgical biopsies and that more than half of them had an infectious contraindication to systemic corticosteroids, notably because they presented with an untreated infection. Conflicting data exist on the interest of low doses of corticosteroids (200 mg/day of hydrocortisone) in ARDS patients. A post hoc analysis [98] of an RCT of low doses of steroids in septic shock patients [99] noted that mortality was improved mainly in the subgroup of patients with ARDS and no response to corticotrophin stimulation. On the other hand, two recent studies suggested that early treatment with low doses of corticosteroids was inefficient or even harmful in patients with H1N1 pneumonia [100] or ARDS [101]. Further randomized, controlled trials are needed before any definitive conclusion.

In the context of a persistent ARDS with histological proof of fibroproliferation, a corticosteroid treatment can be proposed according to the protocol by Meduri et al., with a progressive decrease of doses [95]. This protocol is accompanied by measures usually applied in case of prolonged corticosteroid therapy (gastric protection, prevention of electrolyte disorders, treatment of diabetes, restriction of the use of NMBs) to which can be added a systematic bacteriological monitoring supplemented by other sampling in case of suspicion of infection. One of the main complications of corticosteroid treatment is ICU acquired weakness [54,55], potentially reversible but often leading to difficulties in weaning from ventilation.

## Conclusions

The place of iNO as a rescue therapy has been clarified by numerous studies that gave practical guidelines on its use. The modest reduction of ventilation duration obtained with a conservative fluid strategy for patients who do not have evidence of tissue hypoperfusion makes impossible to create a “gold standard” for management of the fluid status of every ARDS patient. Further studies must confirm recent data from the literature, which provide a strong argument for beneficial effects of NMBs during the early phase of severe ARDS. Concerning corticosteroids, numerous questions remain on their potential benefits in subgroups of ARDS patients. Finally, current and future research has to consider the interest of therapeutics designed to reduce lung inflammation and to enhance alveolar repair. Some of the promising research areas include cell-based therapy with mesenchymal stem cells, which could reverse the major abnormalities of lung injury [102], statin therapy [103], anti-inflammatory drugs, such as aspirin [104], or the modulation of signaling pathway of proteins involved in the regulation of vascular permeability [105,106].

## Competing interests

The authors declare no conflict of interest that may inappropriately influence how results are presented in this paper.

## Authors’ contributions

AR, SH, SD, and LP drafted the manuscript. All authors read and approved the final manuscript.
